# Optimal protamine‐to‐heparin dosing ratio for the prevention of bleeding complications in patients undergoing TAVR—A multicenter experience

**DOI:** 10.1002/clc.23936

**Published:** 2022-10-19

**Authors:** Baravan Al‐Kassou, Verena Veulemans, Jasmin Shamekhi, Oliver Maier, Kerstin Piayda, Tobias Zeus, Adem Aksoy, Andreas Zietzer, Max Meertens, Victor Mauri, Marcel Weber, Jan‐Malte Sinning, Eberhard Grube, Matti Adam, Farhad Bakhtiary, Sebastian Zimmer, Stephan Baldus, Malte Kelm, Georg Nickenig, Alexander Sedaghat

**Affiliations:** ^1^ Department of Medicine II Heart Center, University Hospital Bonn Bonn Germany; ^2^ Division of Cardiology University Hospital of Duesseldorf Duesseldorf Germany; ^3^ Division of Cardiology Cardiovascular Research Institute Duesseldorf Düsseldorf Germany; ^4^ Department of Cardiology, Heart Center University of Cologne Cologne Germany; ^5^ Department of Cardiac Surgery Heart Center, University Hospital Bonn Bonn Germany

**Keywords:** bleeding complication, protamine‐to‐heparin ratio, TAVR, vascular complication

## Abstract

**Background:**

Despite major advances, transcatheter aortic valve replacement (TAVR) is still associated with procedure‐specific complications. Although previous studies reported lower bleeding rates in patients receiving protamine for heparin reversal, the optimal protamine‐to‐heparin dosing ratio is unknown.

**Hypothesis:**

The aim of this study was a comparison of two different heparin antagonization regimens for the prevention of bleeding complications after TAVR.

**Methods:**

The study included 1446 patients undergoing TAVR, of whom 623 received partial and 823 full heparin antagonization. The primary endpoint was a composite of 30‐day mortality, life‐threatening, and major bleeding. Safety endpoints included stroke and myocardial infarction at 30 days.

**Results:**

Full antagonization of heparin resulted in lower rates of the primary endpoint as compared to partial heparin reversal (5.6% vs. 10.4%, *p* < .01), which was mainly driven by lower rates of life‐threatening (0.5% vs. 1.6%, *p* = .05) and major bleeding (3.2% vs. 7.5%, *p* < .01). Moreover, the incidence of major vascular complications was significantly lower in patients with full heparin reversal (3.5% vs. 7.5%, *p* < .01). The need for red‐blood‐cell transfusion was lower in patients receiving full as compared to partial heparin antagonization (10.4% vs. 15.9%, *p* < .01). No differences were observed in the incidence of stroke and myocardial infarction between patients with full and partial heparin reversal (2.2% vs. 2.6%, *p* = .73 and 0.2% vs. 0.4%, *p* = .64, respectively).

**Conclusions:**

Full heparin antagonization resulted in significantly lower rates of life‐threatening and major bleeding after TAVR as compared to partial heparin reversal. The occurrence of stroke and myocardial infarction was low and comparable between both groups.

## Graphical abstract

Optimal protamine‐to‐heparin dosing ratio for the prevention of bleeding complications after TAVR. Full antagonization of heparin was associated with a reduction of life‐threatening bleeding by 69% and major bleedings by 57%, compared to patients with partial heparin reversal. Whereas partial antagonization of heparin was associated with a relative increase of the post‐interventional drop in hemoglobin by 13% and red blood cell transfusion by 53%, as compared to full heparin antagonization. life‐threat., life‐threatening; RBC, red blood cell; TAVR, transcatheter aortic valve replacement.

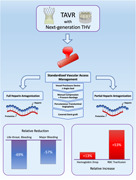

**Abbreviations**
ACT: activated clotting time
AKI: acute kidney injury
CI: confidence interval
IQR: interquartile range
OR: odds ratio
PCI: percutaneous coronary intervention
PTA: percutaneous transluminal angioplasty
STS PROM: thoracic surgeons predicted risk of mortality
TAVR: transcatheter aortic valve replacement
THV: transcatheter heart valve

## 1 INTRODUCTION

Since its introduction in 2002, transcatheter aortic valve replacement (TAVR) has evolved as a key treatment option in interventional aortic valve repair.^1^, ^2^ Several randomized controlled trials showing the benefits of a less‐invasive alternative to surgery have led to a paradigm shift in the treatment of symptomatic severe aortic stenosis, even in patients considered to be at low to intermediate surgical risk.^3^, ^4^, ^5^

Over time, the continuing development of transcatheter heart valve (THV) systems and increasing experience, along with recommendations of clinical best practices, have significantly improved patient outcomes by reducing procedure‐related complications.^6^, ^7^ Nevertheless, TAVR is still associated with several procedure‐specific complications, such as vascular and bleeding events, which have a significant impact on short‐ and long‐term morbidity and mortality.^8^, ^9^, ^10^ Therefore, prevention of bleeding and optimization of the peri‐ and post‐procedural management of vascular complications are paramount to further improvement of patient outcomes following TAVR.

In the context of vascular and bleeding complications, heparin reversal using protamine has been shown to result in significantly lower rates of serious bleeding events in patients undergoing TAVR as compared to those without heparin antagonization.^11^ However, the optimal protamine‐to‐heparin dosing ratio to prevent bleeding complications without increasing ischemic complications in patients undergoing TAVR is unknown. Accordingly, daily clinical practice varies between selective to routine administration of protamine in different dosing ratios, ranging from partial to full antagonization of heparin.

The aim of this observational multicenter study was to compare the safety and efficacy of two different protamine‐to‐heparin dosing ratios for the prevention of bleeding complications in patients undergoing TAVR.

## 2 METHODS

### 2.1 Study population

For the present observational study, we included 1446 patients with severe, symptomatic aortic stenosis undergoing TAVR with the use of next‐generation THV from the RhineHeart TAVR Registry between May 2013 and December 2020.

All included patients were evaluated according to a detailed pre‐TAVR protocol, which included transesophageal echocardiography, multidetector computed tomography, and diagnostic coronary angiography. Reconstructed multidetector computed tomographic images were used for the assessment of aortic valve parameters as well as access vessel diameters and the degree of calcification. Surgical‐risk assessment included consideration of both the EuroSCORE II and the Society of Thoracic Surgeons Predicted Risk of Mortality (STS PROM) estimate. After evaluation, all cases were discussed within the interdisciplinary heart team at the individual centers. In this analysis, patients with a conversion to open heart surgery (*n* = 4) as well as patients undergoing primary surgical cut down for vessel access (*n* = 19) were excluded, as the surgical trauma itself as well as the use of extracorporeal circulation would confound the hemostatic effect of protamine. Written informed consent was obtained from all patients before the procedure. The study complied with Good Clinical Practice guidelines and was approved by the local ethics committees of the individual centers.

### 2.2 Clinical endpoints and follow‐up

The primary endpoint of the study was a composite of 30‐day all‐cause mortality and life‐threatening and major bleeding events. The secondary endpoints included stroke, myocardial infarction, major vascular complications, and acute kidney injury (AKI) at 30 days according to the Valve Academic Research Consortium 3 definition criteria.^12^ Patients were followed‐up during routine outpatient visits and via standardized telephone interviews with the referring general practitioners or cardiologists.

### 2.3 Vascular access management

At the beginning of the procedure, all patients underwent cross‐over angiography to assess the access vessels and to guide the puncture of the common femoral artery. Pre‐closure of the access site was performed using a ProStar or two ProGlide devices, as described previously.^13^ After insertion of the femoral access sheath and before placement of the guiding sheath into the vasculature, all patients received weight‐based unfractionated heparin (70 U/kg) to maintain an activated clotting time (ACT) of 250 to 300 s. After removal of the sheath at the end of the procedure, selective contralateral cross‐over angiography was performed to evaluate the access site for any vascular or bleeding complications. For better comparability of periprocedural vascular injuries, serious extravasation of the access site was documented, which was defined as an effusion ≥5 mm.

According to the standard operating procedure, bleeding prevention started with hemostasis through manual compression for 10 min and subsequent application of a pressure bandage for 12 h. A percutaneous transluminal angioplasty (PTA) was performed for 3 min if noticeable extravasation or bleeding was detectable in the final cross‐over angiography after manual compression. In the case of bleeding that persisted after two PTA attempts, a covered nitinol stent‐graft was implanted. The indication for partial or full antagonization of the heparin with protamine (0.4–0.6 mg vs. 0.9–1.0 mg protamine for each 100 units of heparin given) was left to the discretion of the treating physician, who made the decision according to the angiographic presentation of the access site and the patient's individual thrombotic and bleeding risk. Accordingly, especially patients at increased bleeding risk received full heparin reversal, and toward the end of the study period, as no safety concerns were observed, all patients received full heparin antagonization.

### 2.4 Antithrombotic treatment after TAVR

After the TAVR procedure, continuous intravenous unfractionated heparin was administered in all patients at 100 units/h if the ACT was <40 s to prevent deep vein thrombosis. In the absence of bleeding events, heparin administration was increased to 200 units/h at the second day. In patients with indications for the permanent use of oral anticoagulation, intravenous heparin was given during the first 48 h after the procedure as a bridge to the permanent treatment. In the case of recent a percutaneous coronary intervention (PCI), as well as in patients with aspirin monotherapy, a dual antiplatelet therapy was continued without interruption for at least 3 months.

### 2.5 Statistical analysis

Continuous variables are presented as means with the standard deviation or as medians with an interquartile range (IQR) depending on the distribution, as assessed using the Kolmogorov–Smirnov test. Categorical variables are expressed as absolute numbers and percentages. Comparison analyses of continuous variables were performed using a Student's *t*‐test or Mann–Whitney *U* test according to the distribution. Differences in categorical variables were assessed with a Chi‐squared or Fisher's exact test. Multivariable linear regression analysis was performed to identify independent predictors of cumulative mortality. Hereby, the parameters were first included in a univariate regression analysis. The significant predictors were then entered in a step‐wise multivariate logistic regression model. Statistical analyses were performed with SPSS version 28 (IBM Corporation). Statistical significance was considered as a two‐tailed probability value ≤0.05. All the authors vouch for the accuracy and completeness of the data and analyses.

## 3 RESULTS

The overall study population had a mean age of 81.1 ± 6.0 years with an average STS PROM of 3.6% (IQR: 2.4%–5.5%) and EuroSCORE II of 4.3% (IQR: 2.7%–7.1%), which indicated a low‐ to intermediate surgical risk. Of the patients included, 623 (43.1%) received partial antagonization of heparin and 823 (56.9%) full antagonization of heparin with protamine (Graphic Abstract). The baseline characteristics of the patients, as stratified according to the protamine‐to‐heparin dosing ratio are summarized in Table 1. The average protamine‐to‐heparin dosing ratio was 0.52 ± 0.07 in the partial heparin reversal group. Overall, the clinical variables were well balanced between the two groups, except for lower levels of baseline hemoglobin (11.7 ± 1.8 g/dl vs. 12.3 ± 1.6 g/dl, *p* < .01) and hematocrit (34.9 ± 5.3% vs. 38.0 ± 4.7%, *p* < .001) in patients who received full antagonization of heparin. Moreover, peripheral artery disease was more frequently diagnosed in patients with complete heparin reversal, as compared to patients with partial heparin antagonization (32.6% vs. 19.3%, *p* = .01). However, the mean diameter of the common femoral artery as well as the mean sheath‐to‐femoral artery ratio were comparable between both groups (7.4 ± 2.4 mm vs. 7.1 ± 3.4 mm, *p* = .21; 0.72 ± 0.10 vs. 0.75 ± 0.10, *p* = .07, respectively).

**Table 1:** Baseline characteristics of the patients

	Overall cohort (*n* = 1446)	Partial heparin antagonization (*n* = 623)	Full heparin antagonization (*n* = 823)	*p*‐value
Age, years	81.1 ± 6.0	80.8 ± 6.2	81.3 ± 5.8	.12
Body mass index, kg/m^2^	26.2 (23.6–29.6)	26.4 (23.7–29.6)	26.0 (23.5–29.4)	.17
Female, %	749 (51.8)	344 (55.2)	405 (49.2)	.03
EuroScore II,%	4.3 (2.7–7.1)	4.1 (2.5–7.1)	4.5 (2.8–7.1)	.08
STS‐PROM, %	3.6 (2.4–5.5)	3.4 (2.3–5.6)	3.7 (2.5–5.5)	.07
Left ventricular EF, %	55.7 ± 11.8	55.3 ± 12.0	55.9 ± 11.6	.34
Coronary artery disease, %	917 (63.4)	411 (66.0)	506 (61.5)	.09
Previous PCI, %	519 (35.9)	231 (37.1)	288 (34.9)	.38
Previous cardiac surgery, %	190 (13.1)	76 (12.2)	114 (13.9)	.39
Stroke, %	167 (11.5)	75 (12.0)	92 (11.2)	.62
Diabetes mellitus, %	425 (29.4)	195 (31.3)	230 (27.9)	.18
Peripheral artery disease, %	388 (26.8)	120 (19.3)	268 (32.6)	<.01
Arterial Hypertension, %	1281 (88.6)	554 (88.9)	727 (88.3)	.84
Chronic renal failure, %	733 (50.7)	325 (52.2)	408 (49.6)	.34
Dialysis, %	48 (3.3)	22 (3.5)	26 (3.2)	.77
Creatinine, mg/dL	1.1 (0.9–1.4)	1.1 (0.9–1.4)	1.1 (0.9–1.4)	.11
Hemoglobin, g/dl	12.0 ± 1.8	12.3 ± 1.6	11.7 ± 1.8	<.01
Hematocrit, %	36.3 ± 5.2	38.0 ± 4.7	34.9 ± 5.3	<.01
Platelets, G/l	212 (173–259)	219 (176–264)	208 (170–256)	.07
Highest ACT, sec	257 (229–289)	260 (234–290)	254 (223–288)	.11
Vitamin K antagonists, %	178 (12.3)	79 (12.7)	99 (12.0)	.75
NOAC, %	440 (30.4)	188 (30.2)	252 (30.6)	.86
Dual antiplatelet therapy, %	828 (57.3)	356 (57.1)	472 (57.4)	.95
Common femoral artery, mm	7.3 ± 3.3	7.4 ± 2.4	7.1 ± 3.4	.21
Sheath:femoral artery ratio	0.73 ± 0.10	0.72 ± 0.10	0.75 ± 0.10	.07

Abbreviations: ACT, activated clotting time; EF, ejection fraction; NOAC, non‐vitamin‐K oral anticoagulants; PCI, percutaneous coronary intervention; STS‐PROM, Society of Thoracic Surgeons Predicted Risk of Mortality.

### 3.1 Procedural outcomes

In the overall cohort, TAVR was predominantly performed with the use of the self‐expanding Medtronic Evolut R/PRO prosthesis (59.2%), followed by the balloon‐expandable Edwards SAPIEN 3 prosthesis (29.6%), as shown in Supporting Information: Table 1. Patients receiving full heparin reversal were treated more frequently with an Edwards SAPIEN 3 prosthesis (35.8% vs. 21.3%, *p* < .01), whereas Evolut R/PRO prostheses were more often implanted in patients with partial heparin antagonization (75.6% vs. 46.8%, *p* < .01). In total, pre‐closure of the access vessels was performed by a ProStar device in 35.1% and by two ProGlide devices in 63.4% of the patients. Of note, the ProGlide devices were predominantly used in patients with partial heparin reversal (89.7% vs. 43.5%, *p* < .01), while ProStar devices were more frequently used in patients with full heparin antagonization (56.1% vs. 7.4%, *p* < .01).

In the total study population, periprocedural serious extravasation occurred in 7.7% of cases and was comparable between both groups (*p* = .10). An unplanned bailout endovascular intervention was necessary in 8.7% of patients with partial heparin reversal as compared to 11.3% of patients with full heparin reversal (*p* = .06). Moreover, the mean procedure time and the average amount of contrast dye used was comparable in both groups (70.7 ± 16.8 min vs. 71.1 ± 26.7 min, *p* = .18 and 113.6 ± 28.7 ml vs. 116.2 ± 24.2 ml, *p* = .80; respectively).

### 3.2 Clinical outcomes

**Figure 1:**
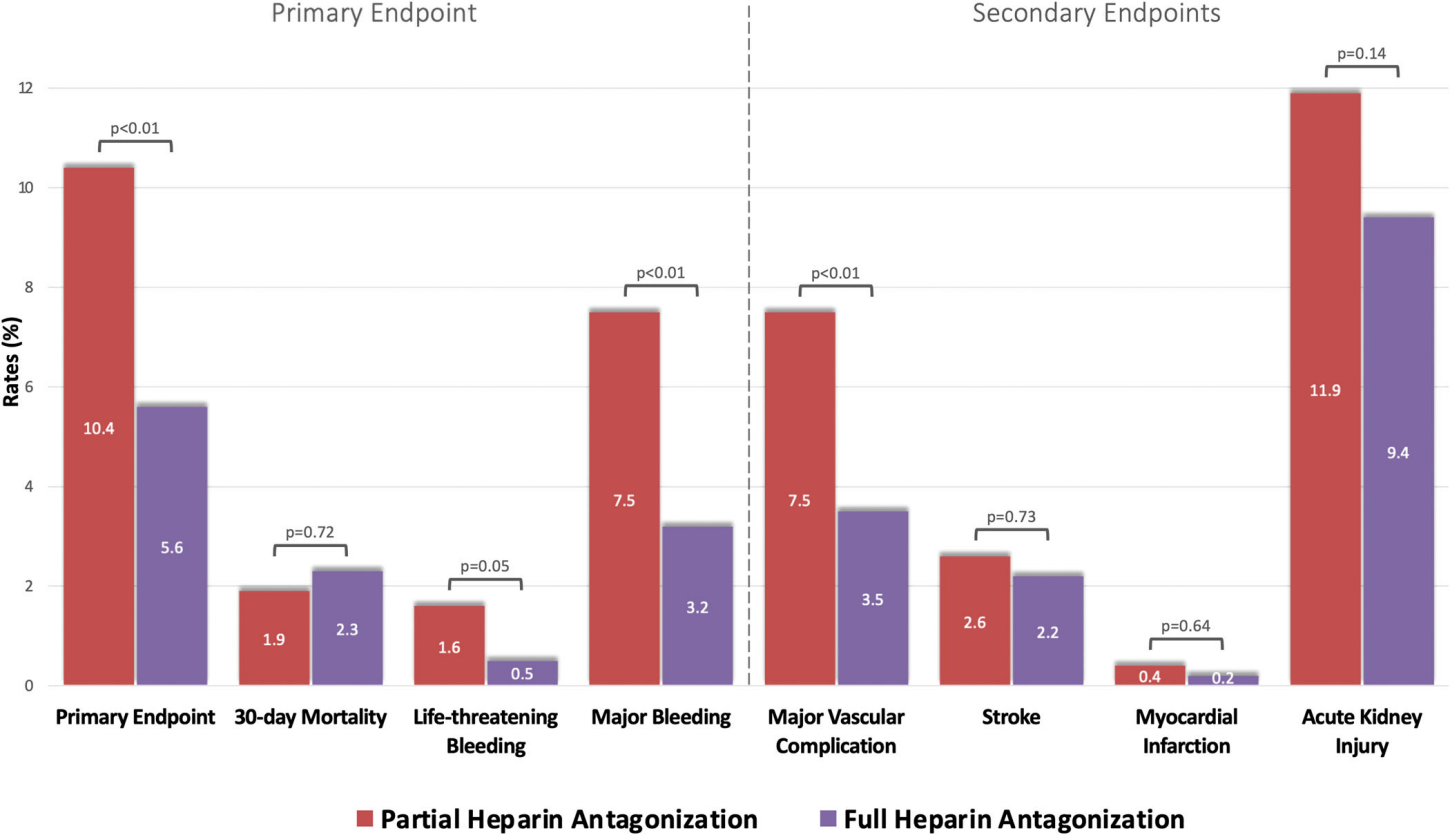
Outcome of patients according to different protamine‐to‐heparin dosing ratios. The primary endpoint, a composite of 30‐day all‐cause mortality, life‐threatening bleeding, and major bleeding, was significantly less frequent in patients with full as compared to partial heparin antagonization, mainly driven by lower rates of bleeding complications in patients with full heparin reversal. Major vascular complications occurred more frequently in patients with partial heparin reversal, whereas there was no difference in the occurrence of stroke or myocardial infarction between the groups.

The primary endpoint of the study, a composite of 30‐day all‐cause mortality and life‐threatening and major bleeding, occurred in 5.6% of the patients with full heparin antagonization, in contrast to 10.4% of the patients with partial heparin reversal (*p* < .01), as presented in Table 2. This difference was mainly driven by lower rates of life‐threatening and major bleeding complications in patients with complete heparin reversal (0.5% vs. 1.6%, *p* = .05; 3.2% vs. 7.5%, *p* < .001, respectively), whereas no significant difference was seen in the 30‐day mortality rates between the groups (2.3% vs. 1.9%, *p* = .72), as displayed in Figure 1. In patients receiving full antagonization of heparin, life‐threatening bleeding resulted in a lethal hemorrhagic shock in two cases, despite appropriate emergency measures being taken. In the partial heparin reversal group, 2 out of 10 cases of life‐threatening bleeding ended fatally, whereas in the remaining cases, the bleeding could be successfully managed. All serious bleeding complications as well as 21 out of 31 (67.7%) deaths within 30 days after TAVR occurred during the in‐hospital stay.

**Table 2:** Clinical endpoints

	Overall cohort (*n* = 1446)	Partial heparin antagonization (*n* = 623)	Full heparin antagonization (*n* = 823)	*p*‐value
Primary endpoint, %	111 (7.7)	65 (10.4)	46 (5.6)	<.01
30‐day mortality, %	31 (2.1)	12 (1.9)	19 (2.3)	.72
Life‐threatening bleeding, %	14 (1.0)	10 (1.6)	4 (0.5)	.05
Major bleeding, %	73 (5.0)	47 (7.5)	26 (3.2)	<.01
Key secondary endpoints				
Major vascular complication, %	76 (5.3)	47 (7.5)	29 (3.5)	<.01
Minor vascular complication, %	304 (21.0)	138 (22.2)	166 (20.2)	.40
Stroke, %	34 (2.4)	16 (2.6)	18 (2.2)	.73
Myocardial infarction, %	4 (0.3)	3 (0.4)	1 (0.2)	.64
Pacemaker implantation, %	181 (12.5)	69 (11.1)	112 (13.6)	.17
Acute kidney injury, %	151 (10.4)	74 (11.9)	77 (9.4)	.14
Red blood cell transfusion, %	185 (12.8)	99 (15.9)	86 (10.4)	<.01

**Figure 2:**
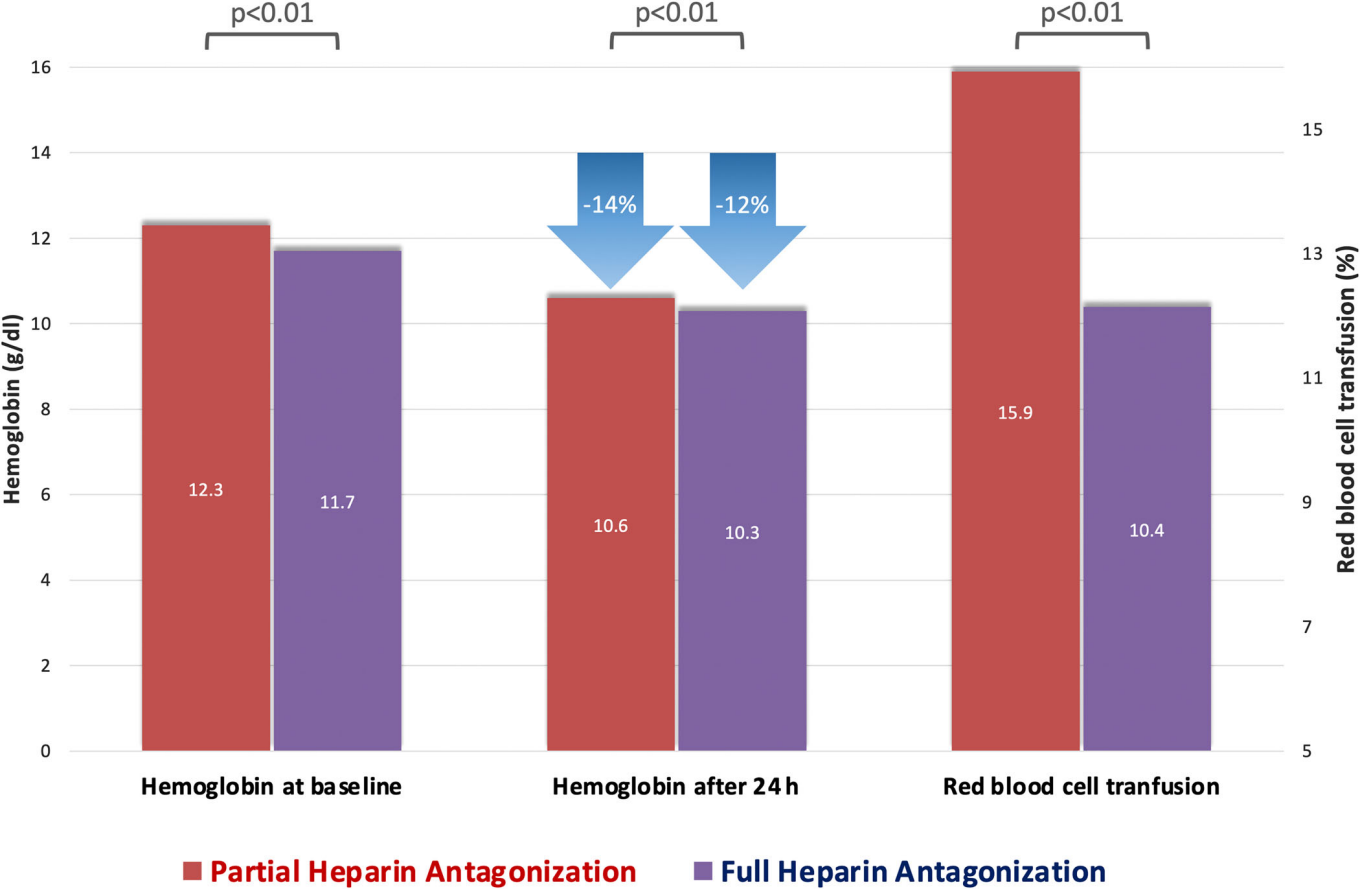
Post‐interventional drop in hemoglobin and the necessity for red blood cell transfusion. Despite a lower hemoglobin level at baseline, patients with full heparin antagonization showed a significantly lower post‐procedural drop in hemoglobin as compared to patients with partial heparin reversal. A red blood cell transfusion was needed more frequently in patients with partial heparin antagonization.

Overall, major vascular complications were observed in 5.3% of cases, with higher rates in patients with partial heparin reversal as compared to patients with full antagonization of heparin (7.5% vs. 3.5%, *p* < .01). However, the incidence of minor vascular complications was comparable between both groups (22.2% vs. 20.2%, *p* = .40). In line with our results on bleeding, the average post‐interventional drop in hemoglobin level was significantly lower in patients with full heparin antagonization in contrast to patients with partial heparin reversal (1.5 ± 1.2 g/dl vs. 1.7 ± 1.2 g/dl, *p* < .01). Consistently, a red blood cell transfusion was needed more frequently in patients with partial antagonization of heparin (15.9% vs. 10.4%, *p* < .01), as shown in Figure 2. Regarding safety endpoints, no difference was detectable in the rates of stroke and myocardial infarction between patients with full as compared to partial heparin reversal (2.2% vs. 2.6%, *p* = .73 and 0.2% vs. 0.4%, *p* = .64, respectively).

### 3.3 Predictors of clinical outcomes

Multivariate regression analyses revealed that only AKI (OR: 2.29 [95% CI: 1.19–4.43], *p* = .01), BMI (OR: 0.93 [95% CI: 0.87–0.99], *p* = .02), and full heparin antagonization (OR: 0.43 [95% CI: 0.24–0.81], *p* < .01) were independently associated with the primary end point, as shown in Table 3. There was no association between pre‐existing peripheral arterial disease (OR: 1.17 [95% CI: 0.76–1.78], *p* = .48) as well as baseline hemoglobin (OR: 0.96 [95% CI: 0.86–1.07], *p* = .48) or hematocrit levels (OR: 1.01 [95% CI: 0.97–1.04], *p* = .77) with the outcome in the univariate analyses. Regarding procedural characteristics, regression analyses showed no association with the primary endpoint between the pre‐closure devices used (OR: 0.78 [95% CI: 0.52–1.18], *p* = .24), the THV implanted (OR: 1.09 [95% CI: 0.80–1.50], *p* = .58), or the contrast dye given (OR: 1.01 [95% CI: 0.99–1.02], *p* = .29).

**Table 3:** Predictors of the primary endpoint

	Univariable analysis			Multivariable analysis		
	*p*‐value	OR	95% CI	*p*‐value	OR	95% CI
STS‐PROM	<.01	1.10	1.03–1.10	‐	‐	‐
EuroSCORE II	<.01	1.10	1.05–1.11	‐	‐	‐
Body mass index	<.01	0.93	0.89–0.98	.02	0.93	0.87–0.99
Vitamin K antagonists	.05	1.63	0.98–2.73	‐	‐	‐
Chronic renal failure	<.01	1.88	1.26–2.82	‐	‐	‐
Coronary artery disease	.05	1.53	1.00–2.36	‐	‐	‐
Hemoglobin after 24 h	<.01	0.60	0.52–0.69	‐	‐	‐
Hematocrit after 24 h	<.01	0.87	0.83–0.91	‐	‐	‐
Procedure time	.01	1.02	1.01–1.02	‐	‐	‐
Acute kidney injury	<.01	3.12	1.95–5.00	.01	2.29	1.19–4.43
Full heparin antagonization	<.01	0.51	0.34–0.75	<.01	0.43	0.24–0.81

Abbreviations: CI, confidence interval; OR, odds ratio; STS‐PROM, Society of Thoracic Surgeons Predicted Risk of Mortality.

## 4 DISCUSSION

The key findings of our observational all‐comers study with a total of 1446 patients undergoing TAVR with next‐generation THVs are:

1. Full antagonization of heparin using protamine resulted in lower rates of the primary endpoint, a composite of 30‐day all‐cause mortality, life‐threatening, and major bleeding complications, as compared to partial heparin reversal.
2. Full antagonization of heparin was safe and did not increase the incidence of thromboembolic events, such as stroke and myocardial infarction.
3. The post‐interventional drop in hemoglobin and the need for red blood cell transfusion was significantly lower in patients receiving full, as compared to partial, heparin antagonization.

Despite considerable advances in the field of TAVR, including continuing technical and clinical developments, vascular and bleeding complications remain a significant procedure‐specific issue, which affects the outcome of patients.^7^, ^9^, ^10^ Although previous as well as current expert consensus documents recommend the consideration of protamine application for heparin reversal to prevent procedure‐related bleeding, there is no recommendation regarding the dosage of protamine in patients undergoing TAVR.^14^, ^15^ Thus, daily practice varies significantly between centers, ranging from complete avoidance of the practice to routine administration of protamine in different doses. In this, the first study evaluating the optimal protamine‐to‐heparin dosing ratio for the prevention of bleeding complications in patients undergoing TAVR, we found that full antagonization of heparin was associated with a 46% reduction of the occurrence of the composite endpoint of 30‐day mortality, life‐threatening, and major bleeding complications, as compared to partial heparin reversal (*p* < .01). This was mainly powered by a reduction of life‐threatening bleedings by 69% and major bleedings by 57% in patients with full antagonization of heparin (*p* ≤ .05). These finding are in line with a recently published study that demonstrated significantly lower rates of serious bleeding events following TAVR in patients receiving protamine for heparin antagonization as compared to patients without heparin reversal.^11^ Moreover, our results are consistent with previous studies of cardiopulmonary bypass and carotid endarterectomy, that reported reduced bleeding complications in patients receiving protamine for heparin reversal.^16^, ^17^, ^18^ Especially in the field of cardiac surgery, evidence is growing, that proper dosing of protamine, to achieve adequate heparin reversal, is superior to under‐ as well as over‐dosing of protamine relative to heparin.^19^, ^20^

With respect to procedure‐related vascular injury, a two‐fold higher incidence of major vascular complications was observed in patients with partial as compared to full antagonization of heparin (*p* < .01). Accordingly, the post‐interventional drop in hemoglobin as well as the need for red blood cell transfusion was 12% and 35% higher in patients with only partial heparin reversal (*p* < .01). From a pathophysiological perspective, this may be explained by the anticoagulant effect of heparin, which indirectly inhibits thrombin and factor Xa by enhancing the activity of the proteinase inhibitor antithrombin III.^21^ This cascade results in a restriction of the formation of fibrin clots at the injury site. Protamine interrupts this process by building inactive complexes with heparin and thus improving hemostasis.^22^

Regarding safety endpoints, the overall incidence of thromboembolic events in this study, including 2.4% stroke and 0.3% myocardial infarction at 30 days, was lower than the rates reported by previously published studies in a comparable cohort of patients, such as the PARTNER II and SURTAVI trial (5.5% and 1.2%; 3.4% and 0.9%, respectively).^3^, ^23^ Nonetheless, our analyses did not reveal any evidence of an increased incidence of thromboembolic events in patients with full as compared to partial heparin antagonization, as the observed rates of stroke (2.6% vs. 2.2%, *p* = .73) and myocardial infarction (0.4% vs. 0.2%, *p* = .64) were comparable between both groups.

Although the present study was observational and not a randomized controlled trial testing the hypothesis under controlled conditions, the findings provide important insights into the real‐life safety and efficacy of the different protamine‐to‐heparin dosing ratios for the prevention of bleeding complications in patients undergoing TAVR.^24^ However, as the data were collected under real‐world conditions, there are some differences between the groups with respect to clinical and procedural characteristics. Patients, who received full heparin reversal had a 40% higher rate of peripheral artery disease and a 5% lower level of hemoglobin at baseline. Moreover, in the overall cohort, pre‐closure of the vessel was mainly performed with ProGlide systems. However, in a head‐to‐head comparison, the ProGlide device was predominantly used in patients with partial heparin reversal, whereas the ProStar device was used more often in patients with full heparin antagonization. This is an important point because some studies indicate that ProStar‐mediated vascular closure is associated with increased rates of serious vascular complications when compared to ProGlide devices.^25^, ^26^ Based on these findings, a beneficial bias in favor of the partial heparin reversal group could be expected. However, in the present study, none of these variables had an impact on the outcome in linear regression analyses. Furthermore, the incidence of periprocedural serious extravasation that required interventional treatment was comparable between the groups (6.6% for partial vs. 8.5% for full heparin antagonization, *p* = .10). In addition, despite the administration of different protamine doses, there was no further difference in the groups with respect to the prevention and management of vascular complications. The rate of unplanned bailout endovascular interventions was comparable between both groups (*p* > .06). Moreover, the procedure time and the amount of contrast dye used did not differ between the groups (*p* > .18). Nonetheless, despite the fact that the patients who received full antagonization were most probably more complex cases and more vulnerable, the outcome was still in favor of the full antagonization group. These findings only underline the superiority of full versus partial heparin antagonization.

When assessing predictors of the outcome, univariate regression analyses showed that higher surgical risk scores, pre‐existing chronic renal failure, and coronary artery disease as well as longer procedure time and an indication for vitamin K antagonists were associated with the occurrence of the primary endpoint. Whereas markers of robust clinical condition, such as high hemoglobin levels and preserved ejection fraction were found to be protective factors in a univariate analysis. However, in multivariate regression analyses, only AKI and BMI remained as independent predictors of the outcome, while full antagonization of heparin remained as the only independent protective predictor of the composite endpoint. These findings are consistent with previous studies reporting an association between the administration of protamine and favorable outcomes in patients undergoing transcatheter cardiac interventions, such as TAVR and PCI.^11^, ^27^ Moreover, the results are in accordance with previously published data showing improved postoperative hemostasis in patients receiving protamine after cardiac surgery.^28^

## 5 LIMITATIONS

The present analysis harbors some important limitations, including the non‐randomized character of our multicenter study. However, to our knowledge, this is the first study evaluating the optimal protamine‐to‐heparin dosing ratio for the prevention of bleeding complications in patients undergoing TAVR. Moreover, although the baseline characteristics were balanced between the two groups, our observational registry data are vulnerable to residual confounding factors. In addition, due to the lack of a recommendation for a standardized protamine‐to‐heparin dosing ratio in our standard operating procedure, patients received protamine at the discretion of the treating physicians, which probably led to a certain selection bias in our analysis. Furthermore, the ACT was not measured routinely in patients after protamine administration to assess the resulting heparin reversal. After the procedure, all patients received continuous low‐dose heparin for thromboprophylaxis for the first 2 days. This should be considered when interpreting the results of this study. However, even though it is likely that the more complex patients at higher bleeding risk might have received the full dose of protamine, the outcome was still in favor of the patients with full heparin antagonization. Nevertheless, an adequately powered randomized controlled trial is needed to verify the findings of this study.

## 6 CONCLUSION

Full antagonization of heparin using protamine after the TAVR procedure was associated with a significantly reduced occurrence of the primary endpoint, as compared to patients with partial heparin reversal. This difference was mainly driven by significantly lower rates of life‐threatening and major bleeding events with full heparin antagonization. Furthermore, the post‐interventional drop in hemoglobin and the need for red blood cell transfusion were significantly lower in patients receiving full, as compared to partial heparin antagonization. Moreover, full antagonization of heparin was safe and did not increase the incidence of thromboembolic events, such as stroke and myocardial infarction.

## CONFLICTS OF INTEREST

Drs. Grube, Sinning, Zimmer, Adam, and Nickenig have received speaker honoraria and research grants from Abbott, Abiomed, Medtronic, Boston Scientific, and Edwards Lifesciences. Dr. Veulemans and Dr. Zeus have received speaker and/or study honoraria from Edwards Lifesciences and Medtronic. Dr. Kelm has received institutional grant support and/or personal fees from Philips, Abbott, Medtronik, Boston Scientific, Mars, Boehringer Ingelheim, Daiichi‐Sanyko GmbH, Amgen, Ancora Heart, and B. Braun. Dr. Grube is a proctor for Boston Scientific and Medtronic. The other authors report no conflicts of interest.

## Supporting information

Supporting information.

## Data Availability

The data that support the findings of this study are available on request from the corresponding author.
