# Supplementary material for: Optimal protamine‐to‐heparin dosing ratio for the prevention of bleeding complications in patients undergoing TAVR—A multicenter experience
Source: Clin Cardiol. 2022 Oct 19;46(1):67–75. doi: 10.1002/clc.23936 (PMC9849438; doi:10.1002/clc.23936)
Supplement: Supplementary file 1 — Supporting information. [file CLC-46-67-s001.docx]

| **Supplemental Table 1: Procedural data** | | | | |
| --- | --- | --- | --- | --- |
|  | **Overall Cohort**  **(n=1446)** | **Partial Heparin Antagonization (n=623)** | **Full Heparin Antagonization**  **(n=823)** | **p Value** |
| Edwards SAPIEN 3, % | 428 (29.6) | 133 (21.3) | 295 (35.8) | <0.01 |
| Evolut R/PRO, % | 856 (59.2) | 471 (75.6) | 385 (46.8) | <0.01 |
| Other valve types, % | 162 (11.2) | 19 (3.0) | 143 (17.4) | <0.01 |
| ProStar, % | 508 (35.1) | 46 (7.4) | 462 (56.1) | <0.01 |
| ProGlide, % | 917 (63.4) | 559 (89.7) | 358 (43.5) | <0.01 |
| Other pre-closure devices, % | 21 (1.5) | 18 (2.9) | 3 (0.4) | <0.01 |
| Serious periprocedural extravasation, % | 111 (7.7) | 41 (6.6) | 70 (8.5%) | 0.10 |
| Unplanned bailout endovasc. interv., % | 147 (10.2) | 54 (8.7) | 93 (11.3) | 0.06 |
| Procedure time, min | 73.8±26.1 | 70.7±16.8 | 71.1±26.7 | 0.18 |
| Contrast dye, mL | 112.5±39.8 | 113.6±28.7 | 116.2±24.2 | 0.80 |
| Hemoglobin after 24 h, mg/dl | 10.4±1.6 | 10.6±1.6 | 10.3±1.6 | <0.01 |
| Hematocrit after 24 h, % | 31.5±4.8 | 32.7±4.7 | 30.3±4.5 | <0.01 |
| Postinterventional hemoglobin drop, g/dl | 1.6±1.2 | 1.7±1.2 | 1.5±1.2 | <0.01 |

*Unplanned bailout endovasc. interv., unplanned bailout endovascular interventions.*
